# Design Strategies for Stack-Based Piezoelectric Energy Harvesters near Bridge Bearings

**DOI:** 10.3390/s25154692

**Published:** 2025-07-29

**Authors:** Philipp Mattauch, Oliver Schneider, Gerhard Fischerauer

**Affiliations:** 1 Chair of Measurement and Control Systems and Center for Energy Technology (ZET), University of Bayreuth, 95447 Bayreuth, Germany; mrt@uni-bayreuth.de; 2 Büro für Strukturmechanik, 96450 Coburg, Germany; oliver.schneider@buerofuerstrukturmechanik.de

**Keywords:** piezoelectric, energy harvesting, finite element, traffic, bridge maintenance, optimization

## Abstract

Energy harvesting systems (EHSs) are widely used to power wireless sensors. Piezoelectric harvesters have the advantage of producing an electric signal directly related to the exciting force and can thus be used to power condition monitoring sensors in dynamically loaded structures such as bridges. The need for such monitoring is exemplified by the fact that the condition of close to 25% of public roadway bridges in, e.g., Germany is not satisfactory. Stack-based piezoelectric energy harvesting systems (pEHSs) installed near bridge bearings could provide information about the traffic and dynamic loads on the one hand and condition-dependent changes in the bridge characteristics on the other. This paper presents an approach to co-optimizing the design of the mechanical and electrical components using a nonlinear solver. Such an approach has not been described in the open literature to the best of the authors’ knowledge. The mechanical excitation is estimated through a finite element simulation, and the electric circuitry is modeled in Simulink to account for the nonlinear characteristics of rectifying diodes. We use real traffic data to create statistical randomized scenarios for the optimization and statistical variation. A main result of this work is that it reveals the strong dependence of the energy output on the interaction between bridge, harvester, and traffic details. A second result is that the methodology yields design criteria for the harvester such that the energy output is maximized. Through the case study of an actual middle-sized bridge in Germany, we demonstrate the feasibility of harvesting a time-averaged power of several milliwatts throughout the day. Comparing the total amount of harvested energy for 1000 randomized traffic scenarios, we demonstrate the suitability of pEHS to power wireless sensor nodes. In addition, we show the potential sensory usability for traffic observation (vehicle frequency, vehicle weight, axle load, etc.).

## 1. Introduction

This contribution aims to describe a combined design methodology of the mechanical and electrical components of a piezoelectric energy harvester near the bearings of roadways bridges and in interaction with the mechanical excitation by the bridge traffic. In addition, dual uses in terms of energy harvesting and sensing (bridge condition monitoring or traffic monitoring) are discussed.

Public roadways are fundamental to both passenger and freight transports [1]. For example, in Germany, close to 80% of all goods are transported by heavy-duty vehicles (trucks) on roadways [2]. Due to the necessity to cross rivers, valleys, or other roads, around 39,500 bridges exist on federal highways in Germany, of which in 2024 only 24.1% manage to achieve a good result regarding quality criteria [3]. To power condition monitoring systems, energy harvesting technologies commonly used in roadway applications are based on solar energy, thermoelectric generators, geothermal energy, and piezoelectric energy harvesting [4].

Piezoelectric transducers as energy harvesting systems (EHSs) have been widely investigated [5,6]. Huang et al. have built an energy-harvesting device for a roadway application, applying a cyclic normal force under laboratory conditions and optimizing the connected load resistor [7]. Zhang et al. modeled a traversing car, but developed an analytical model [8]. Erturk described the theoretical model of a cantilevered beam and a thin piezoceramic patch to harvest the vibrations induced by moving loads [9]. Cahill et al. compared a finite-element (FE) model to differential equations and estimated the energy output of a train passing over a railway bridge. They further validated the results by experimental data obtained from the Skidträsk Bridge in Sweden [10]. Recently, Peralta-Braz et al. proposed a design strategy for cantilever-beam harvesters for bridge applications [11]. Subsequently, Yao et al. improved this model to evaluate the influence of traffic intensity and harvester location [12]. As a rule, the published research focuses on either cantilevered, vibration-based harvesters, working at the host–structure resonance frequency, or stack-based harvesters embedded in the pavement.

Also, the piezoelectric weigh-in-motion systems currently under investigation focus on embedding stack-based piezoelectric sensors in the pavement. Xiong et. al. and Xiang et. al. considered only the sensory aspect and used external data acquisition systems [13,14], whereas Khalili et. al. combined their sensor with an energy harvester and a microcontroller unit for signal processing. However, their main goal was to develop a sustainable method for obtaining roadway traffic data [15]. In contrast, our approach focuses on the design methodology with a sensory dual use in mind.

This work presents an attempt to use stack-based piezoelectric energy harvesters located close to the bridge bearings and absorbing a substantial amount of the dynamic traffic load. The rationale is that such a harvester could simultaneously serve as a sensor that reacts differently to the bridge loads and the bridge condition than a sensor mounted between the bridge bearings and thus provides supplementary information. To the authors’ best knowledge what is missing, however, is a consistent design methodology taking into account the details of the bridge, the piezoelectric harvester, and the dynamic bridge load. The goal of the present work is to present such a methodology.

To determine the forces applied to the energy harvesting system by the traversing vehicles, we have used a three-dimensional FE model. We utilized an approach presented by Hagood et al. to account for the frequency- and therefore also load-dependent stiffness of the electro-mechanical system formed by the shunted piezoelectric harvester [16]. After defining a set of boundary conditions, we use a non-linear optimization algorithm to find the optimal configuration of piezoelectric material and electric load for different traffic scenarios. These scenarios are generated randomly using a set of probability distributions for vehicle frequency, vehicle type, and vehicle weight and are based on real traffic data. The procedure is demonstrated by means of a case study, the bridge over the Callenberger street in Coburg, Germany.

As a result, we have shown that the interaction between the bridge, the harvester, and the expected traffic is crucial for the design of an optimal configuration. Second, our case study shows that the harvested energy is sufficient to power wireless sensor nodes and bridge maintenance sensors discussed in the literature. Furthermore, a detailed examination of the resulting signals shows that the pEHS has the sensing capabilities for traffic observation.

## 2. Materials and Methods

### 2.1. System Structure

**Figure 1:**
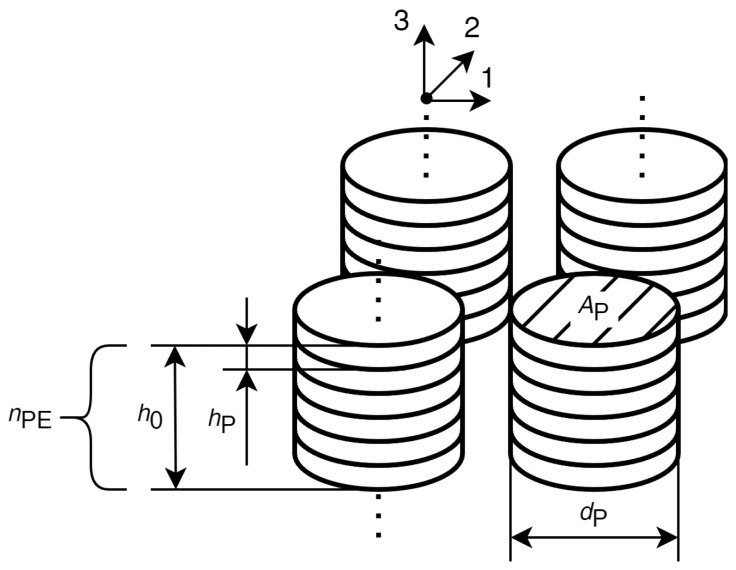
Structure of the presumed EHS.

For all further considerations we will assume an energy harvester structure similar to Figure 1 with $n_{Stacks}$ identical mechanically parallel stacks, each consisting of $n_{PE}$ identical piezoelectric elements that are mechanically connected in series. All piezoelectric elements are electrically connected in parallel, hence the combined internal capacitance of the pEHS $C_{EHS}^{T}$ is the sum of the individual capacitances $C_{P}^{T}$ of the piezoelectric elements

(1) $$\begin{array}{cc} C_{EHS}^{T} & =n_{Stacks}\;\cdot \;n_{PE}\;\cdot \;C_{P}^{T}\\ \; & =n_{PE}^{2}\;\cdot \;\epsilon_{3}^{T}\;\cdot \;\epsilon_{0}\;\cdot \;A_{0}/h_{0}. \end{array}$$

Here, $A_{0}=n_{Stacks}\;\cdot \;A_{p}$ is the total cross-sectional area of the system where $A_{p}$ denotes the area of a single stack, and $h_{0}=n_{PE}\;\cdot \;h_{p}$ is the total height of a stack where $h_{p}$ denotes the height of a single piezoelectric element in the stack. $\epsilon_{3}^{T}$ is the effective relative permittivity at constant stress valid for mechanical loading in the $x_{3}$-direction, and $\epsilon_{0}$ is the permittivity of free space.

We further assume that the force $F_{3}$ acting on each element in the $x_{3}$-direction is identical for all elements in the same stack. However, the force applied to each stack depends on the number of stacks:

(2) $$F_{3}\left(t\right)=F_{EHS}\left(t\right)/n_{Stacks}.$$

when $F_{EHS}$ is the total force on the energy-harvesting system.

A piezoelectric element reacts to mechanical stress by polarization, i.e., a relative shift of charge centers. Owing to non-ideal electrical insulation, the polarization charges will attract free charges of the opposite sign from the environment. One observes a current flow until all polarization charges have been neutralized. Each element in a stack contributes the same amount of current $I_{P}$ to the overall current. Hence,

(3) $$I_{P,EHS}=n_{Stacks}\;\cdot \;n_{PE}\;\cdot \;I_{P}.$$

**Figure 2:**
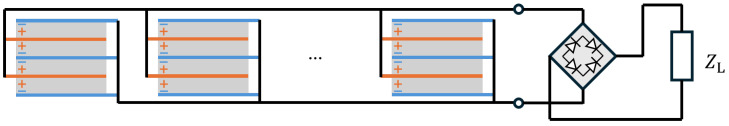
Electric system structure.

The piezoelectric stacks are assumed to be manufactured by stacking individual, disc-shaped elements with alternating polarizations and thin copper electrodes between the discs, as shown in Figure 2. To operate the piezoelectric elements electrically in parallel, every other electrode is connected to the first stack terminal and the remaining electrodes are connected to the second stack terminal.

### 2.2. Mechanical Modelling

#### 2.2.1. Excitation

**Figure 3:**
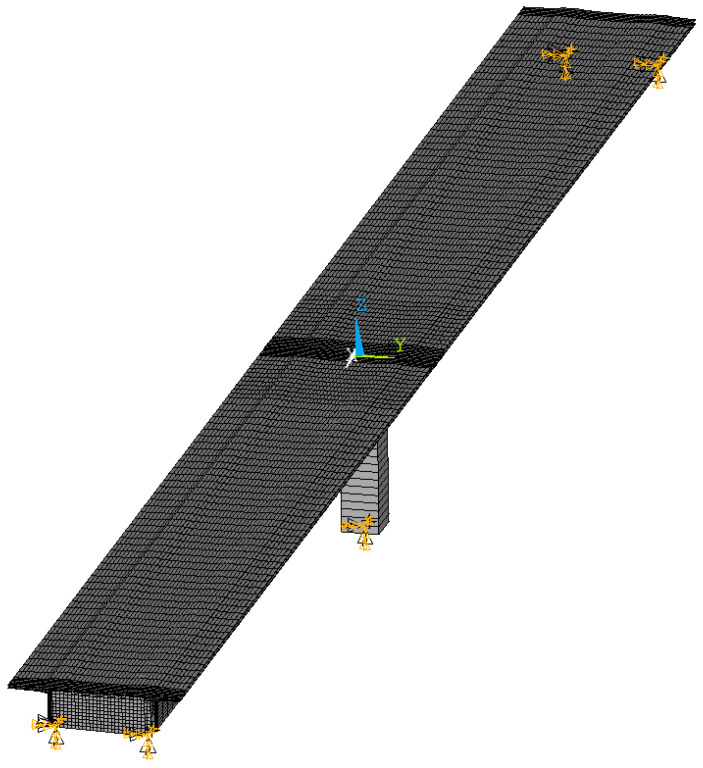
Finite-element model of the bridge over the Callenberger Street in Coburg, Germany, which was used as a test case for our design methodology.

**Figure 4:**
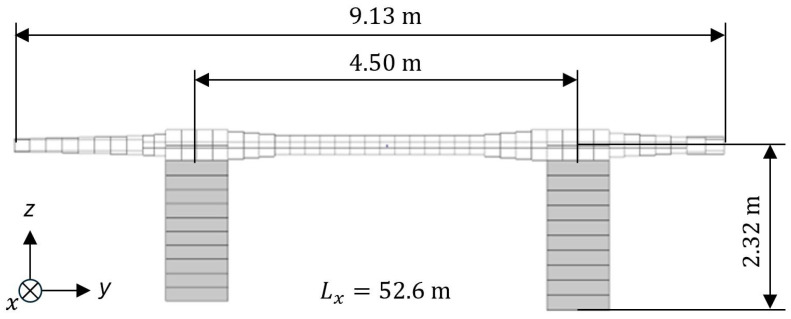
Cross section of the FE model of the bridge over the Callenberger Street in Coburg, Germany.

Figure 3 shows an FE model of the bridge over the Callenberger Street in Coburg, Germany, in Ansys Mechanical APDL. The element formulation SHELL63 is used to recreate the geometry of the bridge out of 2D-shell elements with different thicknesses. The center pillar is defined with the uniaxial BEAM44 formulation using its cross-sectional area. Both the piezoelectric elements of the pEHS with their open-circuit stiffness and the bridge bearings are implemented using the element formulation COMBIN14 for spring-damper elements. Boundary conditions constraining lateral and angular movement have been implemented on the nodes marked in Figure 3. Figure 4 shows a cross-sectional view of the bridge with a width of 9.13 m, a length $L_{x}$ of 52.6 m, and a height from the bearings of 2.32 m. The height of the central pillar shown in Figure 3 is 9.4 m. All shell and beam elements of the bridge are modeled with the material parameters of concrete, as shown in Table 1. The parameters have been experimentally identified from measurement reports according to the German standard DIN 1045-2 [17], and agree with estimations in the literature [18].

**Figure 5:**
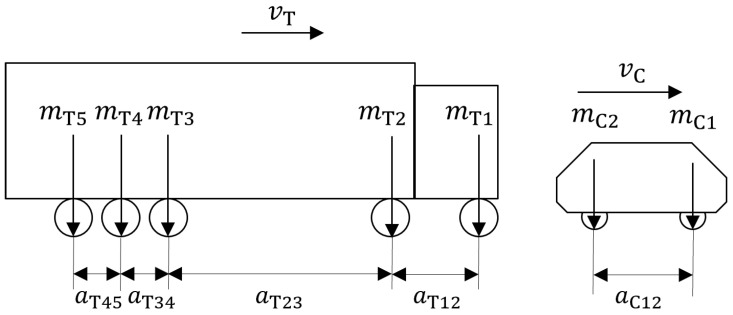
Modelled vehicle geometry.

To describe the traversing vehicles in a discrete-time model, the axle loads are thought to be distributed to all nodes within a search radius of *r* of the axle position. The instantaneous position of the vehicle axles changes within a sampling interval according to the vehicle speed. For simplicity’s sake, we assume that all vehicles drive at constant speed. A total of four load cases have been defined to investigate the effects of different traffic scenarios. Two kinds of vehicles have been modeled: trucks with 5 axles and a total weight of up to 40 tons and regular cars with two axles and a weight of 1.55 tons. In addition, three different loading conditions of the trucks are examined: empty, half-loaded, and fully loaded. Table 2 and Table 3 show the axle distances and the individual axle weights for trucks and cars according to Figure 5. The parameters in Table 2 have been chosen as typical values. The speed of 130 km/h is the target speed on German highways if there is no speed limit. For trucks, the speed limit on German highways is always 80 km/h if there is no lower speed limit. Furthermore, the parameters in Table 3 have been chosen with the European guideline 96/5 3/EC in mind, which limits the vehicle dimensions and axle loads [19].

**Figure 6:**
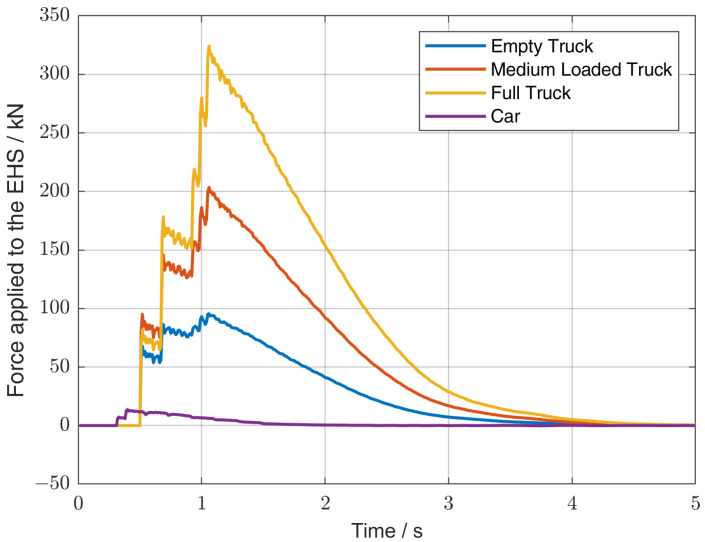
Simulation results of the force acting on a pEHS near the bearing of the Callenberger bridge as a function of time and vehicle type when the stiffness of the piezoelectric material is $5\;\times \;10^{6}\;N\;mm^{-1}$.

In the FE model, the deformation of the COMBIN14 elements making up the pEHS stacks can be evaluated for different load cases and different mechanical stiffnesses of the piezoelectric material. As a result, the mechanical force acting on the pEHS is known and can be used for an optimization of the electrical and mechanical properties of the pEHS. Table 4 lists the general simulation parameters. By way of an example, Figure 6 shows the resulting forces acting on the pEHS according to the mechanical simulation with a stiffness of $5\;\times \;10^{6}\;N\;{mm}^{-1}$ of the piezoelectric material.

#### 2.2.2. Interpolation Based on the Open-Circuit Stiffness

In the iteration process required to find the optimum pEHS design, the geometric properties and the number of piezoelectric elements change with each iteration step. To avoid the necessity to perform a mechanical simulation at each step, the loading force is scaled based on the open-circuit stiffness. The general constitutive equations for piezoelectrics in matrix notation are [20]

(4) $$\begin{array}{cc} D & =\epsilon^{T}E+d\sigma \end{array}$$

(5) $$\begin{array}{cc} S & =d^{T}E+s^{E}\sigma , \end{array}$$

where D and E denote the electric flux density vector and the electric field strength vector. S, σ, and $\epsilon^{T}$ are the second-order tensors of the mechanical strain, mechanical stress, and the electric permittivity under constant stress, respectively. d is the third-order tensor of the piezoelectric charge constants, and $s^{E}$ is the fourth-order elastic compliance tensor under constant electric field strength.

We consider a uniaxial loading case where the normal mechanical stress acts in the same direction in which the piezoelectric element is polarized (the $x_{3}$-direction). In this case, the relationship between external loads and resulting responses is [21]

(6) $$\left[\begin{array}{c} D_{3}\\ S_{3} \end{array}\right]=\left[\begin{array}{cc} \epsilon_{3}^{T} & d_{33}\\ d_{33} & s_{33}^{E} \end{array}\right]\left[\begin{array}{c} E_{3}\\ \sigma_{3} \end{array}\right]$$

where $D_{3}$ and $E_{3}$ are the respective components of the electric flux density and field strength in the $x_{3}$-direction, and $S_{3}$ and $\sigma_{3}$ are the components of the mechanical strain and stress tensors describing uniaxial strain and stress in the $x_{3}$-direction. (We use the common condensed-index notation for all symmetric tensors of order higher than 1.) The quantities $\epsilon_{3}^{T}$, $d_{33}$, and $s_{33}^{E}$ are the relevant components of the permittivity tensor at constant stress, the tensor of piezoelectric charge coefficients, and the compliance tensor at constant electric field strength, respectively (all in condensed-index notation as the tensors are all symmetric). In open-circuit condition, the electric flux density $D_{3}$ is zero, hence we can rearrange the first row of Equation (6) to

(7) $$E_{3}=-\sigma_{3}\;\cdot \;\frac{d_{33}}{\epsilon_{3}^{T}},$$

and insert this into the second row of Equation (6) to obtain

(8) $$S_{3}=\sigma_{3}\;\cdot \;\left(s_{33}^{E}-\frac{d_{33}^{2}}{\epsilon_{3}^{T}}\right).$$

Introducing the electromechanical coupling coefficient $k_{33}=d_{33}/\left(\sqrt{s_{33}^{E}\epsilon_{3}^{T}}\right)$, this becomes

(9) $$S_{3}=\sigma_{3}\;\cdot \;s_{33}^{E}\left(1-k_{33}^{2}\right).$$

Replacing the strain with $S_{3}=\Delta h/h_{0}$ and the mechanical stress with $\sigma_{3}=F_{3}/A_{0}$ and introducing the open-circuit stiffness as $\lambda_{pEHS}^{D}=F_{3}/\Delta h$, we further arrive at

(10) $$\begin{array}{cc} \frac{\Delta h}{h_{0}} & =\frac{F_{3}}{A_{0}}\;\cdot \;s_{33}^{E}\left(1-k_{33}^{2}\right) \end{array}$$

(11) $$\begin{array}{cc} \Leftrightarrow \frac{F_{3}}{\Delta h}=\lambda_{pEHS}^{D} & =A_{0}/\left(s_{33}^{E}\;\cdot \;\left(1-k_{33}^{2}\right)\;\cdot \;h_{0}\right). \end{array}$$

**Figure 7:**
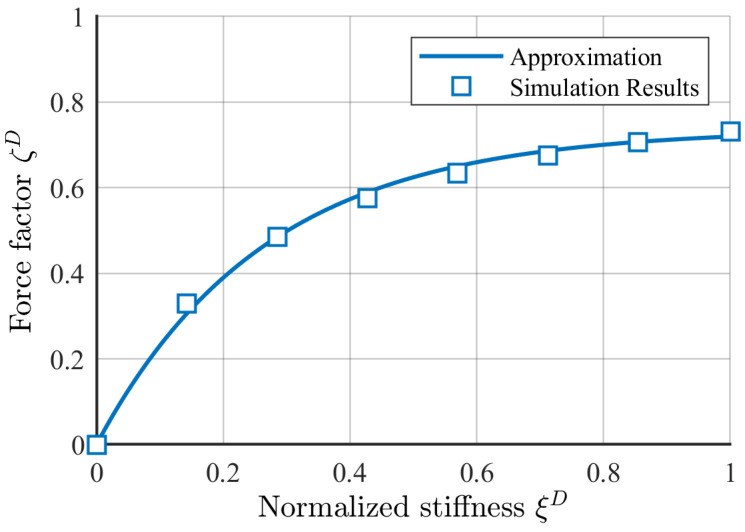
Stiffness-dependent force factor and best-fit curve according to Equation (14).

Here, as before, $A_{0}$ and $h_{0}$ are the total piezoelectric cross section and the stack height, respectively. The normalized stiffness is obtained by

(12) $$\begin{array}{cc} \xi^{D} & =\lambda_{pEHS}^{D}/\lambda_{0}, \end{array}$$

where $\lambda_{0}$ is the highest stiffness used during the simulations. Finally, a force factor $\zeta^{D}$ is introduced as a function of the normalized stiffness $\xi^{D}$ fitting the simulated results by

(13) $$\begin{array}{cc} \zeta^{D} & =f_{k}\;\cdot \;\left(1-\exp\left(-\xi^{D}/a_{k}\right)\right), \end{array}$$

where $f_{k}$ and $a_{k}$ denote the parameters of the exponential fit. Figure 7 shows the interpolated force factor for the simulation results obtained from the Callenberger bridge. The resulting fit curve is

(14) $$\zeta^{D}=0.737\;\cdot \;\left(1-\exp\left(-3.765\;\cdot \;\xi^{D}\right)\right).$$

### 2.3. Electromechanical Modeling

#### 2.3.1. Frequency-Dependent Stiffness

We calculate the frequency-dependent stiffness based on the procedure described by Hagood and von Flotow [16]. The piezoelectric material constants are described in detail in the IEEE Standard on Piezoelectricity [22] and will not be discussed here. We consider a uniaxial loading case where the normal mechanical stress acts in the same direction in which the piezoelectric element is polarized (the ($x_{3}$-direction). For sinusoidal excitations, each quantity can be written as x(t)=ReX_·ejωt with the complex-valued phasor $\underline{X}$ and the angular frequency ω. Under the assumption of uniform sinusoidal fields, we obtain the following expressions for the voltage and current phasors at the terminals of a piezoelectric element in a stack:

(15) $$\begin{array}{cccc} \underline{U} & =\int_{0}^{h_{p}}{\underline{E}}_{3}\;\cdot \;dx={\underline{E}}_{3}\;\cdot \;h_{p} & \; & \Rightarrow {\underline{E}}_{3}=\underline{U}/h_{p}, \end{array}$$

(16) $$\begin{array}{cccc} \underline{I} & =j\omega \left[\int_{A_{p}}{\underline{D}}_{3}\;\cdot \;dA\right]=j\omega {\underline{D}}_{3}\;\cdot \;A_{p} & \; & \Rightarrow {\underline{D}}_{3}=\underline{I}/\left(j\omega A_{p}\right). \end{array}$$

With Equations (15) and (16), we can rewrite Equation (6) as

(17) $$\left[\begin{array}{c} \underline{I}\\ {\underline{S}}_{3} \end{array}\right]=\left[\begin{array}{cc} \epsilon_{3}^{T}/h_{p}\;\cdot \;j\omega A_{p} & d_{33}\;\cdot \;j\omega A_{p}\\ d_{33}/h_{p} & s_{33}^{E} \end{array}\right]\left[\begin{array}{c} \underline{U}\\ \underline{\sigma_{3}} \end{array}\right]=\left[\begin{array}{cc} j\omega C_{p}^{T} & j\omega A_{p}d_{33}\\ d_{33}/h_{p} & s_{33}^{E} \end{array}\right]\left[\begin{array}{c} \underline{U}\\ \underline{\sigma_{3}} \end{array}\right],$$

where $j\omega C_{P}^{T}$ is the internal open-circuit admittance ${\underline{Y}}^{D}\left(j\omega \right)$. Shunting the piezoelectric material adds a parallel admittance ${\underline{Y}}^{SU}\left(j\omega \right)$ to the internal admittance.

(18) $${\underline{Y}}^{Res}\left(j\omega \right)={\underline{Y}}^{D}\left(j\omega \right)+{\underline{Y}}^{SU}\left(j\omega \right).$$

Therefore,

(19) $$\left[\begin{array}{c} \underline{I}\\ {\underline{S}}_{3} \end{array}\right]=\left[\begin{array}{cc} {\underline{Y}}^{Res}\left(j\omega \right) & j\omega A_{p}d_{33}\\ d_{33}/h_{p} & s_{33}^{E} \end{array}\right]\left[\begin{array}{c} \underline{U}\\ {\underline{\sigma }}_{3} \end{array}\right].$$

Our goal is to maximize the power through the shunted circuit, $\underline{I}=0$. Rearranging for the voltage phasor $\underline{U}$ and inserting into Equation (19) yields an equation for the effective frequency-dependent and therefore complex-valued compliance under shunt conditions:

(20) $${\underline{s}}_{33}^{SU}=s_{33}^{E}\;\cdot \;\left(1-k_{33}^{2}\;\cdot \;{\underline{Z}}^{Res}\left(j\omega \right)\;\cdot \;j\omega C_{P}^{T}\right)$$

with ${\underline{Z}}^{Res}\left(j\omega \right)=1/{\underline{Y}}^{Res}\left(j\omega \right)$ and the electromechanical coupling coefficient $k_{33}$. We can then write

(21) $${\underline{S}}_{3}={\underline{s}}_{33}^{SU}\;\cdot \;{\underline{\sigma }}_{3}.$$

The effective compliance ${\underline{s}}_{33}^{SU}$ is different from the purely mechanical compliance $s_{33}^{E}$ and from the (higher) open-circuit compliance. For the shunted case, the effective mechanical stiffness as the inverse of the compliance becomes

(22) $${\underline{\lambda }}_{33}^{SU}\left(j\omega \right)=A_{p}/\left({\underline{s}}_{33}^{SU}\left(j\omega \right)\;\cdot \;h_{p}\right).$$

We will normalize it to the open-circuit stiffness by

(23) $$\begin{array}{cc} {\underline{\bar{\lambda }}}_{33}^{SU} & ={\underline{\lambda }}_{33}^{SU}\left(j\omega \right)/\lambda_{33}^{D}\\ & =\left(1-k_{33}^{2}\right)/\left(1-k_{33}^{2}\;\cdot \;Z^{Res}\left(j\omega \right)/Z^{D}\left(j\omega \right)\right). \end{array}$$

#### 2.3.2. Resistive and Capacitive Shunting

A set of diodes in combination with an additional capacitor $C_{R}$ parallel to the load resistor $R_{L}$ is used to rectify and smooth the output voltage of a pEHS. When the diodes are forward biased, the resulting electric impedance is

(24) $$Z^{Res,RC}\left(j\omega \right)=R_{L}/\left(1+j\omega R_{L}\left(C_{P}^{T}+C_{R}\right)\right).$$

Consequently, the normalized stiffness is

(25) $$\begin{array}{cc} {\underline{\bar{\lambda }}}_{33}^{RC}= & (1-k_{33}^{2})/\\ \; & \left[1-k_{33}^{2}\;\cdot \;j\omega R_{L}C_{P}^{T}/\left(1+j\omega R_{L}\left(C_{P}^{T}+C_{R}\right)\right)\right]. \end{array}$$

However, whenever the rectified voltage is greater or equal to the voltage across the piezo elements, the diodes are reverse biased, and the relevant stiffness is therefore equal to the open-circuit stiffness. Our model does not account for this behavior, as this would make the procedure of calculating the resulting normal forces non-linear. This makes the model conservative in that the force estimated from the pEHS output is underestimated because the open-circuit stiffness always exceeds the shunted stiffness.

#### 2.3.3. Application to Non-Periodic Signals

In practice, the force excitation is not sinusoidal, but any time series can be written as a superposition of sinusoids (Fourier principle). We use a discrete Fourier transformation (DFT) to analyze the individual frequency components of general time signals. In this manner, the time-dependent force exciting an electrically terminated piezoelectric element of a pEHS can be written as

(26) F3(t)=Re∑i=1NF_3i(jωi)·Re{λ¯_33RC(ωi)}·ejωit.

Here, ${\underline{F}}_{3i}\left(j\omega_{i}\right)=A_{i}\left(\omega_{i}\right)\;\cdot \;\exp(j\varphi_{i}\left(\omega_{i}\right)$ is the phasor of the *i*-th force harmonic that would act in the static case and the term Re{λ¯_33RC(ωi)} takes into account the frequency-dependence of the piezoelectric-element stiffness.

#### 2.3.4. Approximation of the Non-Linear Diode Characteristics

**Figure 8:**
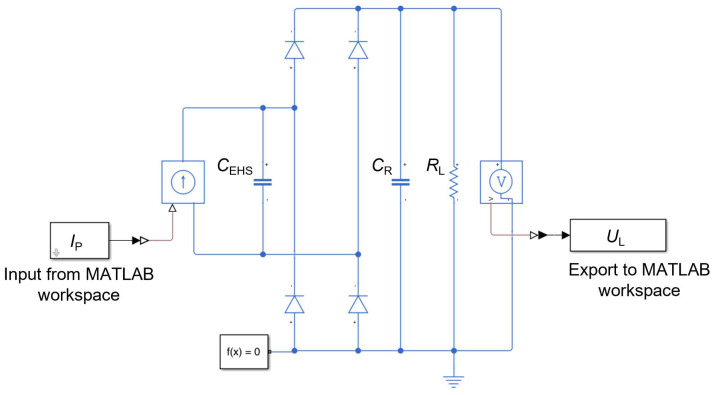
Simulink model used to describe the non-linearity introduced by the diode characteristics.

To take into account the non-linear diode characteristics, we used the Simulink model shown in Figure 8. The input current is the sum of all currents produced by the identical piezoelectric elements connected in parallel:

(27) $$I_{P}\left(t\right)=n_{\mathrm{Stacks}}\;\cdot \;n_{\mathrm{PE}}\;\cdot \;d_{33}\;\cdot \;dF_{P}\left(t\right)/dt.$$

The Simulink model returns the rectified voltage, which is used to calculate the instantaneous power dissipated in the resistive load by Joule’s law and the total amount of dissipated energy:

(28) $$\begin{array}{cc} P_{L}\left(t\right) & =U_{L}^{2}\left(t\right)/R_{L}, \end{array}$$

(29) $$\begin{array}{cc} E_{L}\left(t\right) & =\int_{t}P_{L}\left(t\right)dt. \end{array}$$

### 2.4. Statistical Variation

**Figure 9:**
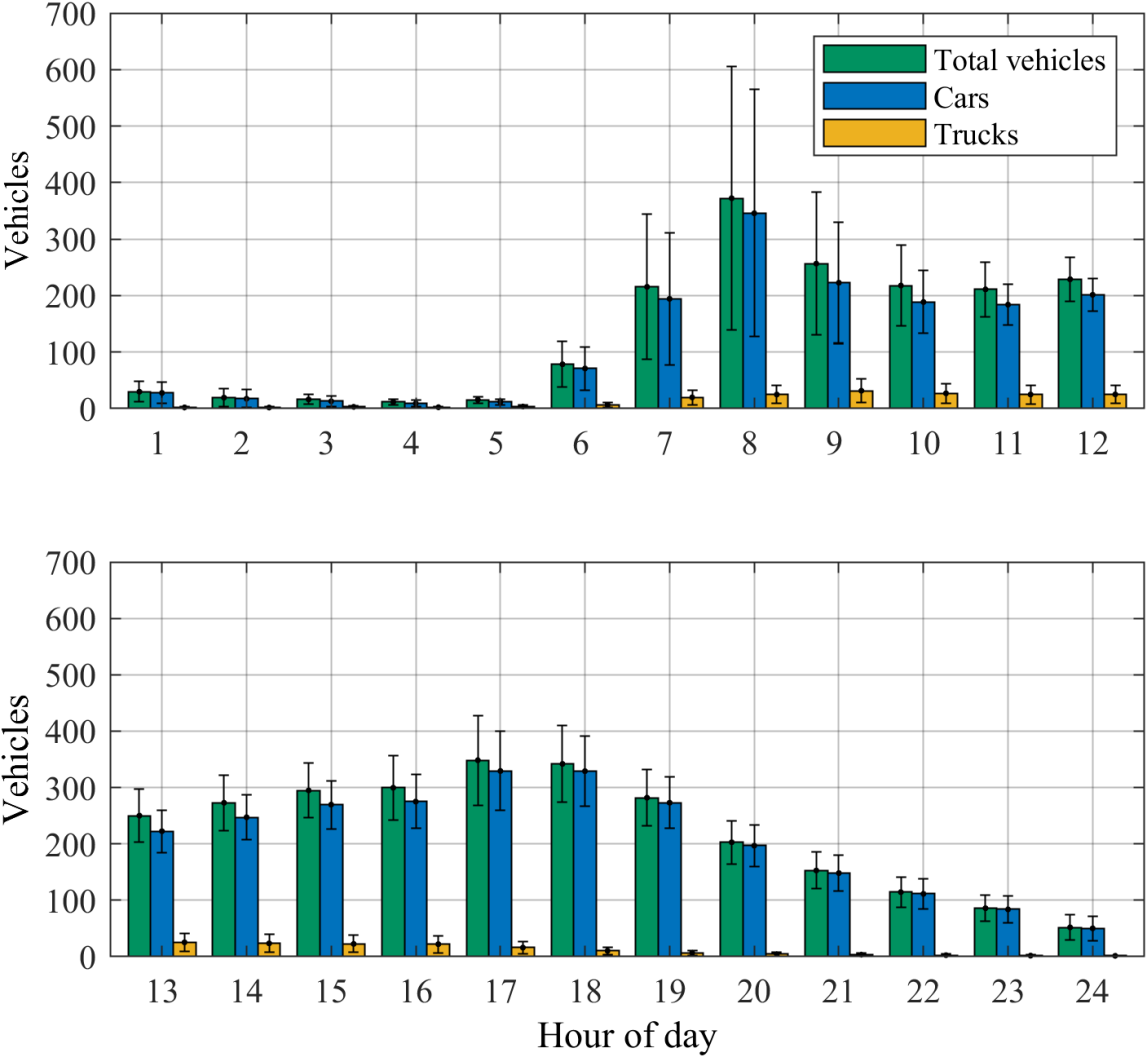
Vehicles per hour of the day, averaged over one year at the traffic counting station 9237 in the direction of Coburg. The error bars represent the standard deviation. (Raw data from [23]).

A traffic census allowed us to obtain the traffic distribution over the year on an hourly basis (Figure 9) [23].

**Figure 10:**
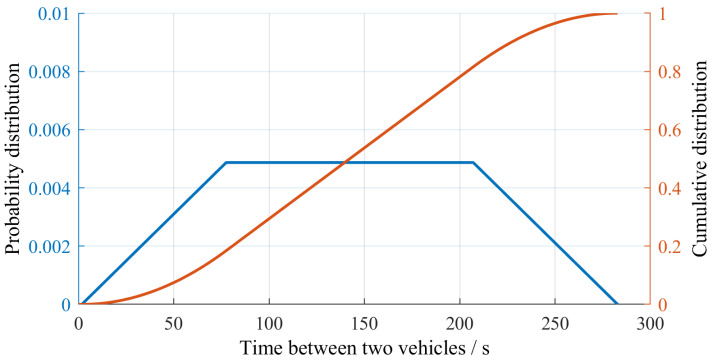
Trapezoidal probability distribution for the time at which a vehicle crosses the Callenberger bridge within any of the time intervals of duration $t_{1}=282.8$ s within the 1st hour of the day (hour between 12 p.m. and 1 a.m.).

Lacking statistical evidence for the distribution of vehicles within any given hour of the day, we assumed a uniform distribution. Given the average number ${\bar{N}}_{v,i}$ of vehicles in the *i*-th hour of the day, we divided the hour into intervals of duration $t_{i}=1\;h/{\bar{N}}_{v,i}$. Vehicles are expected to keep a minimum safety distance equal to 0.5·v/1000·1 h where *v* denotes the vehicle speed. This condition may be violated in practice once in a while, but such occasional violations have a negligible effect on the validity of our calculations. The condition of minimum distance was taken into account by assuming a trapezoidal probability distribution function for the occurence of a vehicle in each of the time intervals of duration $t_{i}$ (Figure 10). The plateau duration was chosen as twice the standard deviation of the $t_{i}\;\cdot \;\sigma_{N,i}/{\bar{N}}_{v,i}$ with $\sigma_{N,i}$ the observed standard deviation of the number of vehicles in the *i*-th hour of the day over the year. Finally, the vehicle type assumed to cross the bridge in any of the intervals of duration $t_{i}$ was determined at random based on the ratio of trucks to passenger vehicles with a normal distribution for both trucks and passenger vehicles.

**Figure 11:**
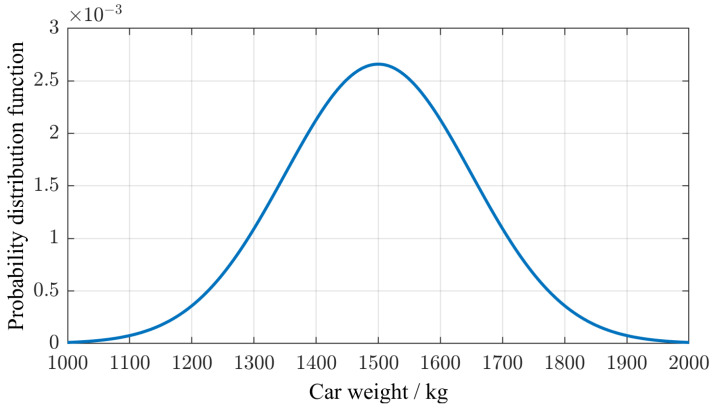
Assumed distribution of the weights of passenger vehicles.

The vehicle weights were scaled by a normally distributed random factor with a mean of 1 and a standard deviation of 0.1 (Figure 11).

**Figure 12:**
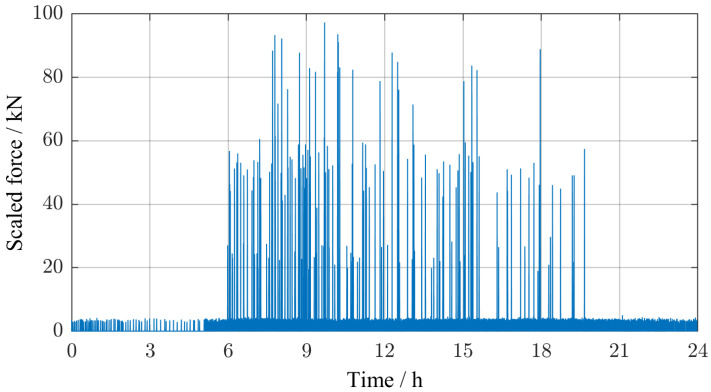
Example of a randomly generated combined excitation signal, scaled by the frequency-dependent stiffness factor. See text for an explanation of the synthesis algorithm.

The synthetic excitation signal consisted of many individual load profiles. Starting at 12 p.m., the time lapse until the next vehicle crossing was generated based on the described trapezoidal probability distribution and the type of the next vehicle was determined at random. Figure 9 suggests that there is almost no truck traffic between 8 p.m. and 6 a.m. on the Callenberger bridge. Consequently, during this time of the day, only the load profiles of passenger vehicles were considered in the synthetic excitation signal. At the start of each optimization process, a set of individual days was created and then used during each iteration. An example excitation signal, scaled by the frequency-dependent stiffness factor, is shown in Figure 12.

### 2.5. Boundary Conditions for the Case Study on the Callenberger Bridge

**Figure 13:**
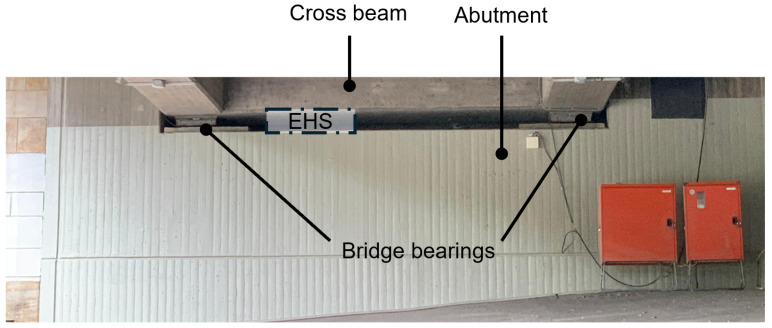
Available installation space under the Callenberger bridge.

**Figure 14:**
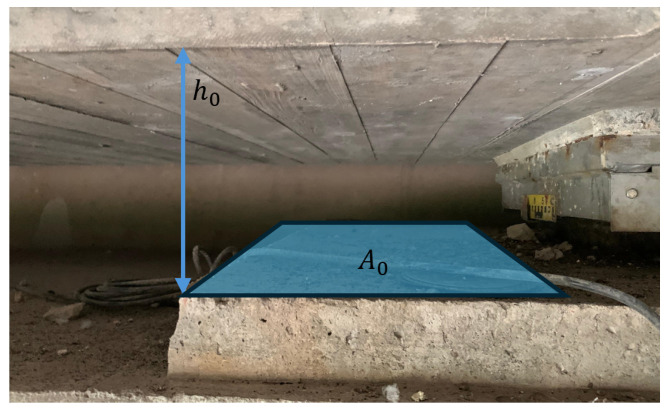
Detail view of installation space under the Callenberger bridge.

Figure 13 and Figure 14 show the available space for the pEHS between the crossbeam and the bridge abutment. The minimum piezoeletric element thickness $h_{P,min}$ is arbitrarily chosen as 1.0 mm. Due to the limited installation space, the geometric dimensions are restricted to

(30) $$\begin{array}{cc} h_{0} & \leq \;200.0\;mm \end{array}$$

(31) $$\begin{array}{cc} A_{0} & \leq \;0.1\;m^{2} \end{array}$$

(32) $$\begin{array}{cc} n_{PE} & \leq h_{0}/h_{P,min} \end{array}$$

In addition, we assume the stiffness of the bridge bearings to be

(33) $$\begin{array}{cc} \lambda_{XY} & =375000\;N\;{\mathrm{mm}}^{-1} \end{array}$$

(34) $$\begin{array}{cc} \lambda_{Z} & =750000\;N\;{\mathrm{mm}}^{-1} \end{array}$$

These stiffnesses have been determined with load deflection measurements according to the German guideline for the recalculation of existing road bridges [24].

Abramovitch et al. have shown that a mechanical stress level of up to 30 MPa can be considered a safe-side stress level and reported a significant decrease in the piezoelectric properties for stresses above 50 MPa. Therefore, to avoid a depolarization and reduced piezoelectric properties, an upper threshold for the acceptable surface pressure has been defined. For all further considerations, we assume a mechanical construction that limits the mechanical loading to this threshold of $\sigma_{th}\;=\;30\;M\mathrm{Pa}$ [25].

We demonstrate the method with the material constants in Table 5, which describe the typical and commercially available materials Sonox P5 and Sonox P502 from the manufacturer CeramTec (Plochingen, Germany) [26].

### 2.6. Optimization Algorithm

The optimization was implemented in MATLAB [27] through the function fmincon, an algorithm that searches for a (local) minimum in a constrained function with multiple variable parameters. The goal was to find a combination of piezoelectric-element stacks, defined by the size and the amount of stacks, and electric embedding that leads to a maximized output based on realistically defined boundary conditions and excitations. The target function to be minimized is the inverse of the energy transferred to the load resistor for a given traffic scenario. The degrees of freedom of the optimization algorithm are:

- The stack height $h_{0}$,
- the total piezoelectric cross-sectional area $A_{0}$,
- the number of elements per stack $n_{PE}$,
- the number of stacks per pEHS $n_{Stacks}$,
- the load resistance $R_{Load}$, and
- the rectifying capacitance $C_{Rect}$.

In each iteration cycle, the following steps are executed:

1. Create a new set of input parameters.
2. Scale the force signal based on the new open-circuit stiffness.
3. Apply the frequency-dependent stiffness scaling in the frequency domain.
4. Reassemble the force signal and calculate the current from the equivalent current source.
5. Solve the Simulink model for the rectified voltage.
6. Compute the total converted energy at the load resistor for the chosen traffic scenario.

## 3. Results and Discussion

### 3.1. Time-Series Evaluation of the Optimized Configuration

Applying the algorithm to the Callenberger bridge yielded the results summarized in Table 6.

**Figure 15:**
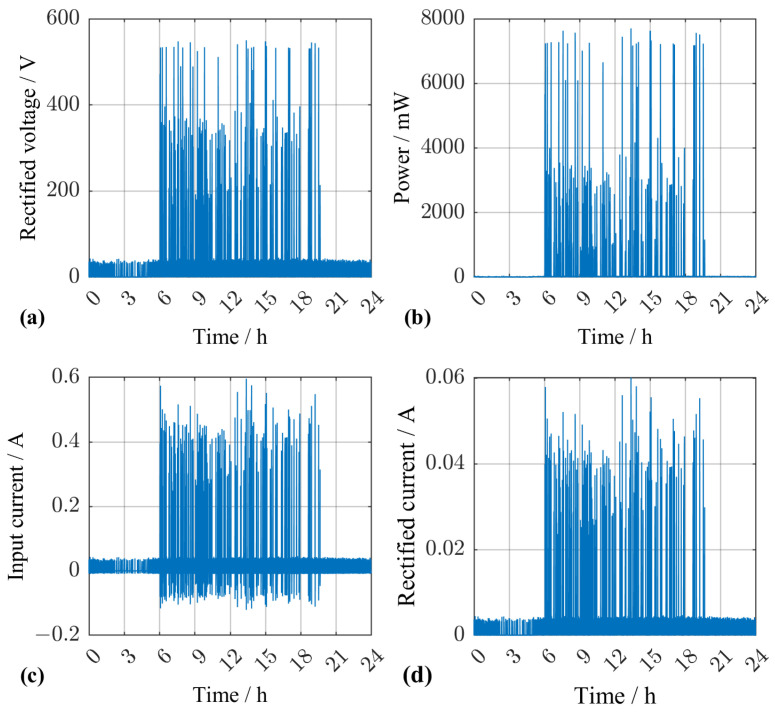
Simulation results for a randomly chosen traffic scenario. (**a**) Rectified voltage at the capacitor $C_{R}$ and the load resistor $R_{L}$. (**b**) Converted power at the load resistor $R_{L}$. (**c**) Input current resulting from the force exciting the pEHS. (**d**) Current flowing though the rectifying stage to the load.

Figure 15a shows the time-dependent voltage across the capacitor $C_{R}$ and the resistor $R_{L}$ over one day for a randomly chosen traffic scenario. The time constant $\tau =C_{R}\;\cdot \;R_{L}$ is small enough to make every individual vehicle distinguishable with passenger vehicles leading to voltages of around 40 V and trucks, depending on their weight, leading to 200 V–550 V. Due to this voltage difference, the converted power at the load resistor (Figure 15b) is only negigibly influenced by passenger-vehicle traffic. A comparison of Figure 15c,d reveals that, due to the high total capacitance of the pEHS, only around 10% of the input current flows through the rectifying stage.

The average power harvested with this configuration was in the order of milliwatts, which suffices to power modern bridge condition monitoring sensors [28,29]. In combination with the latest ultra-low power microcontrollers and energy-efficient wireless communication protocolls such as LoRa, the pEHS could then serve as an autonomous sensor node [30,31].

**Figure 16:**
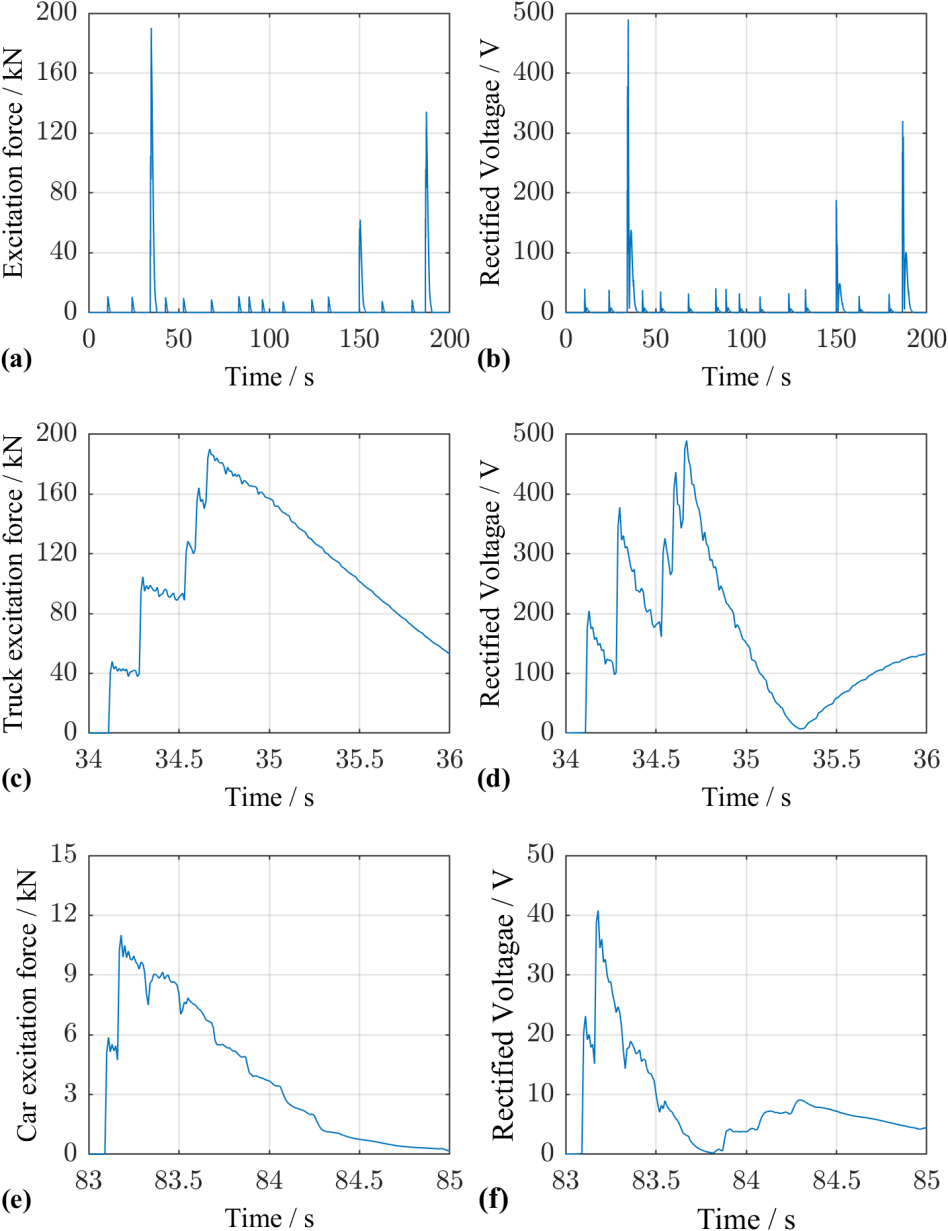
Excitation force from vehicle crossing and resulting rectified voltage for a specific traffic scenario. (**a**) Excitation forces from trucks and cars over a duration of 200 s. (**b**) Rectified voltage in response to the forces in (**a**). The vehicles are clearly distinguishable. (**c**) Time segment with excitation force of a 5-axis truck. (**d**) Rectified voltage in responste to the force in (**b**), with peaks corresponding to each of the 5 axles. (**e**) Time segment with the excitation force of a car with two axles. (**f**) Rectified voltage in response to the force in (**e**), with peaks for each car axle.

The comparison of the excitation signal in Figure 16a and the resulting rectified voltage in Figure 16b demonstrates that passing vehicles can be distinguished. Figure 16c–f show details for time intervals in which a five-axle truck and a passenger vehicle, respectively, pass the bridge. For both vehicle types, local peaks in the time response can be attributed to the action of individual vehicle axles.

### 3.2. Measure to Reduce the Time Variation of the EHS Output Voltage

**Figure 17:**
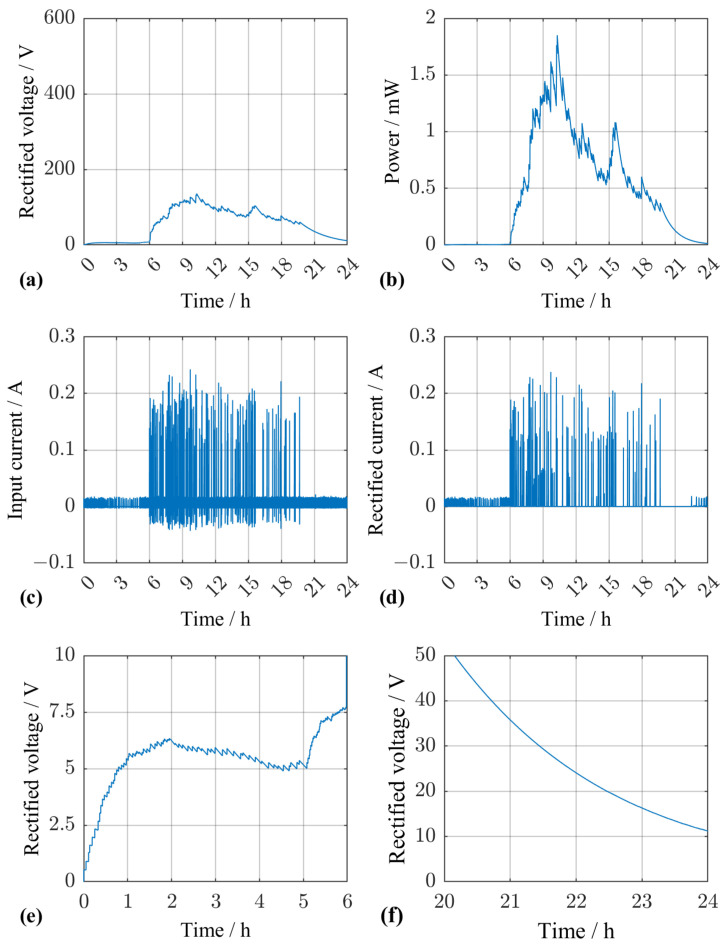
Simulation results for a randomly chosen traffic scenario with modified load stage. (**a**) Rectified voltage across the capacitor $C_{R}$ and the load resistor $R_{L}$. (**b**) Converted power at the load resistor $R_{L}$. (**c**) Input current resulting from the excitation of the pEHS. (**d**) Current flowing though the rectifying stage to the load. (**e**) Rectified voltage from midnight to 6 a.m. (**f**) Rectified voltage from 8 p.m. until midnight.

To achieve a more stable output voltage, e.g., to use a step-down converter to bring the voltage down to battery voltage level, we could increase the rectifying capacitance or the load resistance (or both). We repeated the simulation to investigate the impact on the output power, but arbitrarily changed the capacitance to 1.0 mF and the resistance to 10 MΩ. The results are visualized in Figure 17. In the night, between midnight and 6 a.m., passenger vehicles lead to a stable voltage level of around 6 V. During the daytime the voltage level mainly depends on the trucks (Figure 17a). Figure 17e,f show the voltage from midnight to 6 a.m. and from 8 p.m. to midnight, respectively, in detail. At the start of the simulation the rectifying capacitor was assumed to be empty; hence, the voltage at midnight is 0 V. The rectifier output voltage then rises and stays between 5 V and 7 V depending on the traffic details in the time interval between midnight and 6 a.m. Figure 17b shows that the converted power does not fluctuate strongly throughout the day. Due to the much higher load resistance, the power peaks are drastically reduced compared to Figure 15b. Likewise, a comparison of Figure 17c,d reveals that almost all of the input current is transferred through the rectifying stage because the capacitance $C_{R}$ now exceeds the inertial capacitance of the EHS $C_{EHS}$ by far. In addition, passenger vehicles no longer contribute to the current flow once the voltage rises above a certain threshold. In Figure 17d, they start contributing again at 10:30 p.m. when the voltage drops below 20 V. The amount of charge transferred, however, is too small to stop the capacitor from discharging back to the stable voltage level seen in Figure 17a. This phenomenon occurs whenever the force exciting an individual piezoelectric element does not suffice to raise its open-circuit voltage above the instantaneous voltage $U_{L}\left(t\right)$ across the capacitance $C_{R}$, i.e., whenever

(35) $$d_{33}\;\cdot \;F_{P}\left(t\right)/C_{P}^{T}=d_{33}\;\cdot \;F_{EHS}\left(t\right)/\left(n_{Stacks}\;\cdot \;C_{P}^{T}\right)\leq U_{L}\left(t\right).$$

### 3.3. Statistical Evaluation

**Figure 18:**
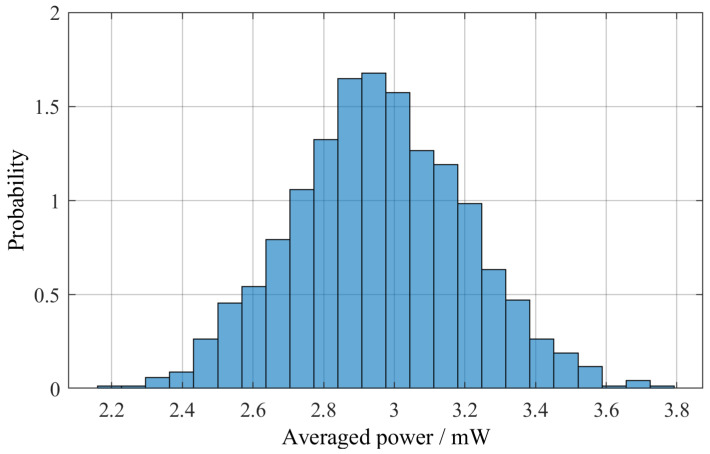
Empirical probability density distribution of the averaged power over one day, observed in a numerical experiment with N = 1000 random traffic scenarios.

After determining the optimal configuration, we created 1000 synthetic different traffic scenarios with the method described above to observe the statistical properties of the resulting system. The figure of merit used to compare the individual scenarios is the average output power of the pEHS over one day, i.e., the total amount of energy converted over one day divided by 24 h. This procedure yielded the histogram, or empirical probability density distribution, shown in Figure 18. The distribution is characterized by a mean value of 2.96 mW and a standard deviation of 0.25 mW, equalling 8.36% of the mean value.

## 4. Conclusions

Based on the combination of mechanical simulation, analytic calculations and the numerical solution of non-linear component equations, we have demonstrated a method to obtain an optimal pEHS configuration for a inclusion in a custom bridge. Here, “optimal configuration” means that a change in the choice of piezoelectric material, the geometry of the piezoelectric stacks or the electrical load of the harvester would lead to a decrease in the power harvested from the given bridge excited by the given traffic.

The case study on the Callenberger bridge also showed that the day-by-day variation of traffic causes a standard deviation of the power output of below 10% compared to the mean value. The average power harvested with this configuration was in the order of milliwatts, which suffices to power modern bridge condition monitoring sensors. As the Callenberger bridge is a relatively small bridge without high traffic volume, an optimized pEHS mounted near the bearings of highway bridges with high truck traffic is expected to yield a much higher power output.

Designing the pEHS for a small time constant τ would result in strongly time-varying output voltages with high spikes alternating with idle phases. As we have shown, the voltage response can be smoothed by increasing τ, but one advantage of small time constants is still worth mentioning: such a pEHS is suitable for gathering information on the traversing vehicles based on the magnitude of the rectified voltage.

Finally, we mention that our investigation has focussed on the optimum pEHS design for a given environment (bridge, bearing, and traffic). In practice, one would need additional circuitry and an energy management system to power one or more sensor devices.

It should also be noted that off-the-shelf commercial devices that match the requested properties may not be available. However, industrial companies like CeramTec offer customized piezoelectric elements that can be stacked within the factory. Furthermore, depending on the conditions on site, it can be difficult to obtain the data required for optimization. This is especially true for older bridges that need to be modernized. Unknown or uncertain traffic information can lead to over- or underengineering of the pEHS. In addition, a pEHS in an optimized configuration might not make sense from a technical-economic point of view, e.g., if the expected traffic volume is too low to justify the expensive piezoelectric elements.

Future research could include the implementation of an energy management system (EMS) and a battery model in the simulation to calculate the charging states. Further integration of a virtual model of a microcontroller would add realistic load conditions to the model. Another point that should be addressed in future research is the design of a suitable overload protection that is compatible with the often harsh conditions and high geometric tolerances on bridges.

Our further goal is to combine the pEHS with such an EMS and a wireless sensor node and to demonstrate the system performance in a field environment.

## Figures and Tables

**Table 1:** Material parameters used for the concrete elements.

Model Parameter	Symbol	Value
Young’s modulus	$Y_{\mathrm{Concrete}}$	32.84 GPa
Shear modulus	$G_{\mathrm{Concrete}}$	13.68 GPa
Poisson’s ratio	$\nu_{\mathrm{Concrete}}$	0.2
Mass density	$\rho_{\mathrm{Concrete}}$	$2500\;kg\;m^{-3}$

**Table 2:** Parameters used to model passenger vehicles.

Model Parameter	Symbol	Value
Car velocity	$v_{C}$	$130\;km\;h^{-1}$
Distance axle 1–axle 2	$a_{C12}$	2.5 m
Axle load 1	$m_{C1}$	0.775 t
Axle load 2	$m_{C2}$	0.775 t

**Table 3:** Parameters used to model trucks.

Model Parameter	Symbol	Value		
Velocity	$v_{T}$	$80\;km\;h^{-1}$		
Distance axle 1–axle 2	$a_{T12}$	3.73 m		
Distance axle 2–axle 3	$a_{T23}$	5.61 m		
Distance axle 3–axle 4	$a_{T34}$	1.30 m		
Distance axle 4–axle 5	$a_{T45}$	1.30 m		
Loading case		Empty	Medium	Full
Load axle 1	$m_{T1}$	7.00 t	9.81 t	8.43 t
Load axle 2	$m_{T2}$	3.00 t	6.79 t	11.39 t
Load axle 3	$m_{T3}$	1.00 t	3.31 t	6.39 t
Load axle 4	$m_{T4}$	1.00 t	3.31 t	6.39 t
Load axle 5	$m_{T5}$	1.00 t	3.31 t	6.39 t

**Table 4:** Simulation parameters of the mechanical simulation using Ansys Mechanical.

Model Parameter	Symbol	Value
Minimum element length	$\Delta l_{min}$	0.193 m
Time Step	Δt	0.01 s
Search Radius	*r*	0.5 m

**Table 5:** Piezoelectric material parameters.

Parameter	Symbol	Value
Relevant piezoelectric charge coefficient	$d_{33}$	$440\;\times \;10^{-12}\;CN^{-1}$
Relevant mechanical compliance tensor component	$s_{33}^{D}$	$19\;\times \;10^{-12}\;m^{2}N^{-1}$
Relevant permittivity tensor component	$\epsilon_{33}^{T}/\epsilon_{0}$	1800

**Table 6:** Results of the optimization.

Parameter	Symbol	Value
Cross-sectional area	$A_{0}$	$4900\;mm^{2}$
Stack height	$h_{0}$	195 mm
Number of stacks	$n_{Stacks}$	7
Elements per stack	$n_{PE}$	195
Smoothing capacitance	$C_{Rect}$	1.35 μF
Load resistance	$R_{Load}$	39.3 kΩ

## Data Availability

The data are accessible online: https://rdspace.uni-bayreuth.de/handle/rdspace-ubt/351 (accessed on 27 July 2025).
